# Metaproteomic Profile of the Colonic Luminal Microbiota From Patients With Colon Cancer

**DOI:** 10.3389/fmicb.2022.869523

**Published:** 2022-04-14

**Authors:** Alessandro Tanca, Marcello Abbondio, Giovanni Fiorito, Giovanna Pira, Rosangela Sau, Alessandra Manca, Maria Rosaria Muroni, Alberto Porcu, Antonio Mario Scanu, Paolo Cossu-Rocca, Maria Rosaria De Miglio, Sergio Uzzau

**Affiliations:** ^1^Department of Biomedical Sciences, University of Sassari, Sassari, Italy; ^2^Medical Research Council (MRC), Centre for Environment and Health, Imperial College London, London, United Kingdom; ^3^Department of Pathology, Azienda Ospedaliero-Universitaria di Sassari, Sassari, Italy; ^4^Department of Medical, Surgical and Experimental Sciences, University of Sassari, Sassari, Italy; ^5^Surgical Pathology Unit, Department of Diagnostic Services, “Giovanni Paolo II” Hospital, Area Socio-Sanitaria Locale (ASSL) Olbia-Azienda per la Tutela della Salute (ATS) Sardegna, Olbia, Italy

**Keywords:** colon lumen, colorectal cancer, gut microbiota, metaproteome, tumor-infiltrating lymphocytes

## Abstract

Recent studies have provided evidence of interactions among the gut microbiota (GM), local host immune cells, and intestinal tissues in colon carcinogenesis. However, little is known regarding the functions exerted by the GM in colon cancer (CC), particularly with respect to tumor clinical classification and lymphocyte infiltration. In addition, stool, usually employed as a proxy of the GM, cannot fully represent the original complexity of CC microenvironment. Here, we present a pilot study aimed at characterizing the metaproteome of CC-associated colonic luminal contents and identifying its possible associations with CC clinicopathological features. Colonic luminal contents were collected from 24 CC tissue specimens immediately after surgery. Samples were analyzed by shotgun metaproteomics. Almost 30,000 microbial peptides were quantified in the samples, enabling the achievement of the taxonomic and functional profile of the tumor-associated colonic luminal metaproteome. Upon sample aggregation based on tumor stage, grade, or tumor-infiltrating lymphocytes (TILs), peptide sets enabling discrimination of sample groups were identified through discriminant analysis (DA). As a result, *Bifidobacterium* and *Bacteroides fragilis* were significantly enriched in high-stage and high-grade CC, respectively. Among metabolic functions, formate–tetrahydrofolate ligase was significantly associated with high-stage CC. Finally, based on the results of this pilot study, we assessed the optimal sample size for differential metaproteomic studies analyzing colonic luminal contents. In conclusion, we provide a detailed picture of the microbial and host components of the colonic luminal proteome and propose promising associations between GM taxonomic/functional features and CC clinicopathological features. Future studies will be needed to verify the prognostic value of these data and to fully exploit the potential of metaproteomics in enhancing our knowledge concerning CC progression.

## Introduction

The development of colorectal cancer (CRC) is a complex and heterogeneous process, involving both genetic and epigenetic alterations, as well as other relevant factors, such as diet, exposition to microbes, and host immunity (Kuipers et al., 2015). For many years, CRC diagnosis and prognosis have been based exclusively on clinicopathological criteria, building on histopathological classifications and tumor staging systems. The application of molecular genetics methodologies has widened the knowledge concerning the CRC pathogenesis, providing bases for new molecular classifications and for the identification of more accurate prognostic and predictive indicators (Nguyen and Duong, 2018). Current trends recognize increasing prognostic meaning to the tumor microenvironment, with specific focus on the antitumoral immune response, including tumor-infiltrating lymphocytes (TILs) (Taube et al., 2018). Functional morphology analyses enabled a more in-depth evaluation of relative frequencies of lymphoid subpopulations and led to conceive an “immunoscore,” which proved to be more effective than the tumor-node-metastasis (TNM) staging as a prognostic parameter (Pagès et al., 2018).

The gut microbiota (GM) is constituted by a huge number of microbial species living close to the colorectal epithelium and is able to regulate key physiological processes, including immune response and metabolism (Wong and Yu, 2019). Several large metagenomic studies discovered associations between specific GM signatures and the colon adenoma-carcinoma sequence, providing evidence for the key role of the GM in the evolution of CRC (Nakatsu et al., 2015; Tilg et al., 2018). In particular, a strong and positive association between the abundance of *Fusobacterium nucleatum* and CRC severity has been observed (Brennan and Garrett, 2019; Lee et al., 2019). It is also known that the interaction between the GM and local host immune cells can lead to regression or progression of intestinal tumors (Leman et al., 2020). Furthermore, food composition is largely recognized as a key risk factor for CRC. In addition, diet is known to affect gut health *via* its effects on GM metabolism, which in turn influences host immunity, gene expression and epigenetic modulation (O’Keefe, 2016).

Among the “omic” approaches employed to analyze the GM, metaproteomics has been demonstrating its ability to take a detailed picture of the biological functions carried out by the members of complex microbial communities in a given health or disease state (Tanca et al., 2017a; Heintz-Buschart and Wilmes, 2018). In a recent, pilot metaproteomic study, Long et al. (2020) compared the fecal metaproteome between newly diagnosed patients with CRC and healthy controls, finding 341 microbial proteins (mainly related to iron intake/transport, oxidative stress, and DNA replication) with significantly different abundance between the two sample groups. Although reporting interesting and promising results, this study did not investigate the possible correlations between GM functions and CRC stratification based on site, grade, or stage, and did not provide information concerning the relationship between TILs and microbial proteins. In addition, although stool can be considered a reasonable proxy of the GM, it cannot fully reproduce the original complexity of the tumor-associated colon microenvironment; even more importantly, microbial communities can rapidly modify their expression patterns in response to several environmental stimuli occurring while passing through the distal colon and remaining in the rectal ampulla, as well as in the time between evacuation and sample collection (Tanca et al., 2017b; Tang et al., 2020).

In view of the above, we performed a pilot study based on the metaproteomic characterization of the tumor-associated colonic luminal contents collected from 24 patients with colon cancer (CC). The aims of this pilot study were: (i) to functionally characterize the microbiota of colonic luminal content samples collected during CC surgery by metaproteomics; (ii) to investigate the associations between metaproteomic features (taxa and functions) and the clinicopathological features of CC (stage, grade, and TILs) using several statistical approaches, including discriminant, enrichment, and correlation analyses; (iii) to carry out a power analysis aimed at estimating the sample size needed to find robust, statistically supported results when employing a metaproteomic approach.

## Materials and Methods

### Samples

The study was conducted in accordance with the code of ethics of the World Medical Association (Declaration of Helsinki). The study protocol was reviewed and approved by the Bioethics Committee of the “Azienda Sanitaria Locale di Sassari” (n. 2032/CE, 13/05/2014), and written informed consent was obtained from each patient.

Patients with histological diagnosis of CC subjected to surgical resection in the Surgery Unit of the Sassari University Hospital from June 2014 to December 2015 were included in the study. Exclusion criteria were hereditary CRC, other malignancy, multiple tumor recurrence, previous radiotherapy or chemotherapy treatment, severe diarrhea, incomplete clinicopathological information about the tumor, and scarce amount of colonic luminal material. Patients were not subjected to any preoperative bowel preparation. Colonic luminal contents overlaying the resected lesion (generally comparable to solid stool according to their texture and appearance) were collected under sterile conditions in the operatory room immediately after surgery. Based on the inclusion/exclusion criteria, 24 samples were considered for this pilot study.

All samples were immediately frozen and stored at −20°C until use. Then, samples were thawed at 4°C to collect two equal portions (weighing approximately 150 mg each), with the first being subjected to protein extraction for (meta)proteomic analysis and the second to DNA extraction for metagenome sequencing.

Surgical specimens were processed according to histopathology procedures, and hematoxylin and eosin (H&E)-stained slides were analyzed by an experienced pathologist to achieve a final diagnosis of CC histotype and differentiation grade, according to the criteria of the World Health Organization (Bosman et al., 2010). Tumor staging was performed according to the American Joint Committee on Cancer (AJCC) Staging Manual (Amin et al., 2017). Further clinicopathological data were obtained from medical records.

The assessment of TILs was determined by two independent pathologists on H&E slides, based on the recommendations by the International TILs Working Group, as previously described (Fuchs et al., 2020).

### Metaproteome Analysis

Protein extraction from colonic luminal content samples was performed as described earlier for fecal samples (Tanca et al., 2014). Accordingly, samples were resuspended in an extraction buffer (2% SDS, 100 mM DTT, 20 mM Tris-HCl pH 8.8) and incubated at 95°C for 20 min in a thermoblock (FALC, Treviglio, Italy). After adding a steel bead (5 mm diameter; Qiagen, Hilden, Germany) to each sample, the samples were sequentially incubated at −80°C for 10 min, subjected to bead beating for 10 min (30 cycles/s in a TissueLyser LT mechanical homogenizer, Qiagen), incubated at −80°C for 10 min, then at 95°C for 10 min, subjected to bead beating for 10 min (30 cycles/s), and centrifuged at 20,000 × g for 10 min. The supernatant was collected as a protein extract.

Protein extracts were processed according to a modified filter-aided sample preparation (FASP) protocol (Wisniewski et al., 2009; Tanca et al., 2013). Accordingly, the protein extracts were diluted with UA solution (8 M of urea in 100 mM of Tris-HCl, pH 8.8), loaded onto an Amicon Ultra-0.5 filtration device (30 kDa cutoff; Merck, Darmstadt, Germany) and centrifuged at 14,000 × g for 15 min. Then, 200 μl of UA solution, 100 μl of 50 mM iodoacetamide in UA solution, 100 μl of UA solution, additional 100 μl of UA solution, and 100 μl of 50 mM ammonium bicarbonate were sequentially added to the sample, followed by centrifugation. Finally, trypsin (1 μg in 50 mM ammonium bicarbonate solution) was added to each sample, followed by incubation at 37°C overnight. Peptide mixtures were collected by centrifugation; a final elution with 100 μl elution solution (20% acetonitrile, 0.2% formic acid) was also performed. Peptide mixtures were concentrated and resuspended in 0.2% formic acid.

Liquid chromatography-tandem mass spectrometry (LC-MS/MS) analyses were carried out in service using a Q-Exactive Orbitrap mass spectrometer (Thermo Fisher Scientific, Waltham, MA, United States), operating with an EASY-spray source, interfaced with an Easy-nLC 1000 LC system (Thermo Fisher Scientific). Peptide mixtures were concentrated and desalted using StageTips, in-house made according to the protocol described by Rappsilber et al. (2007). Then, peptides (load range 1–2 μg per run) were separated by LC with a C18 EASY-spray column (PepMap RSLC C18, 75 μm × 500 mm, 2 μm, 100 Å, Thermo Fisher Scientific) at 35°C with a flow rate of 250 nL/min for 135 min, using the following three-step gradients of eluent B (0.2% formic acid in 95% acetonitrile) in eluent A (0.2% formic acid in 5% acetonitrile): 1–30% for 115 min, 30–60% for 10 min, and 60–95% for 10 min. Samples were run in a randomized order and a blank run was carried out after each sample. The mass spectrometer was set up in a data-dependent MS/MS mode under direct control of the Xcalibur software (version 4.1.31.9), where a full scan spectrum (from 300 to 1,700 m/z) was followed by the MS/MS spectra. The instrument was operated in a positive mode. The temperature of the ion transfer capillary, the spray voltage, and the S-lens RF level were set to 250°C, 1.6 kV, and 50, respectively. The mass spectra were acquired with full MS mode at a resolution of 70,000 within a mass range of 300–1,700 m/z, with 1.0 × 10^6^ of automatic gain control (AGC) target and 120 ms of maximum ion injection time. After ion activation/dissociation, the 12 most abundant peaks (Top 12 method) were measured with higher energy C-trap dissociation at a normalized collision energy of 25%. The MS/MS spectra acquisition was carried out with a resolution of 35,000, with 5.0 × 10^5^ of AGC target and 120 ms of maximum ion injection time. Dynamic exclusion was set to 30 s and nitrogen was used as the collision gas.

Peptide identification was performed using Proteome Discoverer (version 2.4; Thermo Fisher Scientific), with Sequest-HT as the search engine and Percolator for peptide validation, setting the false discovery rate (FDR) threshold to 1%. Search parameters were as follows: precursor mass range 350–3,500 Da; minimum peak count 5; S/N threshold 1.5, enzyme trypsin; maximum missed cleavage sites 2; peptide length range 6–50 amino acids; precursor mass tolerance 10 ppm; fragment mass tolerance 0.02 Da; static modification cysteine carbamidomethylation; and dynamic modification methionine oxidation. Searches were conducted in parallel against 3 sequence databases: (i) a collection of metagenomic sequences obtained in-house from a pool of the study samples (generated as described in the section ‘‘Generation of a Custom Metagenomic Database’’; 147,265 sequences in total); (ii) a public human gut metagenome dataset^1^ (9,878,647 sequences in total) (Li et al., 2014); (iii) a human proteome database (retrieved from UniProtKB/Swiss-Prot, release 2019_06; 42,420 sequences in total). The identified peptides matching with sequences belonging to the first two databases used in this study (i.e., custom and/or public human gut metagenomes) were pre-classified as “microbial” and subjected to a further taxonomic filtering (Unipept assignment to Archaea or Bacteria, see below). The identified peptides matching with sequences belonging to the human database were classified as “human.”

The “Spectrum Files RC” node was used to perform offline mass recalibration, while the “Miinora Feature Detector” node was used for label-free MS1 quantitation. After mass recalibration and feature alignment, the optimal settings for the determination of retention time and mass tolerance windows were calculated by the Minora algorithm, based on the distribution of mass accuracy and retention time variance. A consensus feature list was created by the software based on the outputs of the “Feature Mapper” and “Precursor Ions Quantifier” nodes. For all peptides significantly matching with at least an MS2 spectrum in at least one sample, the corresponding MS1 signals were mapped across runs and then quantified by taking the maximum peak intensity. Peptide intensities were considered as abundance measure. The abundance of a given (taxonomic or functional) feature was estimated by summing the intensity values of all peptides having that feature among their annotations.

The Unipept web application (v.4.0)^2^ was used to carry out taxonomic annotation (*via* the lowest common ancestor classification) of the identified peptide sequences (Gurdeep Singh et al., 2019). Protein sequences matching with at least an MS spectrum with FDR > 1% (217,360 sequences) were subjected to functional annotation by alignment against a database containing all bacterial sequences from UniProtKB/Swiss-Prot (release 2019_09) using DIAMOND (blastp module, *e*-value threshold 10^–4^); Kyoto Encyclopedia of Genes and Genomes (KEGG) orthologous group (KOG) information was then retrieved from the UniProt website *via* the “retrieve” tool based on the corresponding accession numbers (UniProt Consortium, 2019). Among the different types of functional annotation available in UniProt, the KOG annotation was chosen as less ambiguous than the “Protein name” annotation and more specific than the “Protein family” annotation. Furthermore, proteins with no KOG annotation but sharing the same protein name (according to UniProt) were given a custom functional annotation (having a code starting with “CKO”), in order to minimize the functional information loss.

### Generation of a Custom Metagenomic Database

The DNA was extracted from colonic luminal content samples with the QIAamp Fast DNA Stool Mini Kit (Qiagen). DNA quantification was carried out using a Qubit™ Fluorometer with the dsDNA High Sensitivity assay kit (Life Technologies, Carlsbad, CA, United States, now Thermo Fisher Scientific). A DNA pool was prepared by mixing an equal volume of DNA extract from each sample to carry out a shotgun sequencing of the whole gut metagenome. The DNA was subjected to tagmentation and ligation of MiSeq adaptors according to the instructions of the Nextera XT kit (Illumina, San Diego, CA, United States). Libraries (average size of 500 bps) were validated by capillary electrophoresis on a chip using the BioAnalyzer 2100 instrument with the High Sensitivity DNA Kit (Agilent Technologies, Santa Clara, CA, United States), quantified with the Qubit dsDNA High Sensitivity assay kit, and finally normalized. Libraries were sequenced in service using a MiSeq sequencer (Illumina). The MiSeq Reagent Kit v3 from Illumina was used (following the manufacturer’s specifications) to generate paired-end reads of 201 bases in length in each direction.

Raw reads were either filtered and clustered without assembly or assembled into contigs. In the first case, read processing was carried out using fastq_mergepairs (parameters: fastq_truncqual 3, fastq_minovlen 8), fastq_filter (parameters: fastq_truncqual 15, fastq_minlen 205), and cluster_smallmem (identity threshold 1) tools from the VSEARCH suite v.2.13.6 (Rognes et al., 2016). In the second case, read assembly into contigs was carried out using MetaVelvet v.1.2.02 (Namiki et al., 2012), by setting 61 as k-mer length, 200 as insert length, and 300 as minimum contig length. Open reading frame (ORF) finding was carried out using FragGeneScan v.1.31 (Rho et al., 2010), training for Illumina sequencing reads with about 0.5% error rate. Clustered reads and assembled contigs (amino acid sequences) were appended in a single fasta file, which was subjected to removal of redundant sequences using CD-HIT (Fu et al., 2012) and used as sequence database for peptide identification (refer to the section “Metaproteome Analysis”).

### Statistical Analyses

Alpha diversity was calculated as richness and Shannon’s index (Shannon, 1948). For each sample, richness was defined as the number of non-zero abundance peptides, whereas Shannon’s index was computed using the formula *H* = ∑_*i*_pi*ln(*pi*) where *p*_*i*_ is the relative abundance of the ith peptide in a sample. To deal with the different number of peptides measured in different samples, we defined pi*ln(*pi*) = 0 for all peptides with abundance equal to zero. Differences among groups (defined based on gender, age, tumor site, tumor stage, and TILs, as illustrated in Supplementary Figures 4, 5) were tested using Wilcoxon rank sum or Kruskal–Wallis non-parametric tests for patients’ or tumor characteristics with two or more categories, respectively.

Beta diversity among the groups was evaluated by performing the principal component analysis (PCA) on peptide intensity data using the web application ClustVis^3^ (Metsalu and Vilo, 2015). To calculate the proportion of variability in the whole metaproteomic dataset explained by each clinical variable, we applied the principal component partial R-square (PCPR2) method (Fages et al., 2014). Briefly, this method is composed of two main steps: (1) for each clinical variable and each eigenvector of the PCA, the mutual *R*^2^ parameter (proportion of variance explained) is computed from a one-way ANOVA test; (2) for each clinical variable, the proportion of variability explained on the whole dataset is calculated as the sum of the *R*^2^ parameters weighted by the PCA eigenvalues.

Discriminant analyses (DAs) were performed on log-transformed peptide intensities using sparse partial least squares regression (sPLS) implemented in the Bioconductor package mixOmics (v.6.14.1) (Rohart et al., 2017). Briefly, sPLS performs variable selection and integration into a single step (based on LASSO regression) to maximize the covariance between the biomarker matrix and the clinical variable to discriminate. The optimal analysis parameters (number of components and number of peptides) were selected through a cross-validation procedure implemented in the mixOmics package. We repeated the analysis 1,000 times, splitting the dataset into training set and test set to identify the optimal set of discriminating the peptides and reduce the overfitting as much as possible. Finally, we identified those peptides selected at least in 50% of the dataset permutations. The classification/discrimination performance was evaluated *via* hierarchical clustering based on the Euclidean distance (complete linkage method) and represented through heatmaps. Accuracy and area under the curve (AUC) were used as classification metrics. Accuracy was computed as the number of correct predictions out of the total; AUC was calculated as the average of 20 training cross-validation sets (80–20% split at each iteration), as implemented in the mixOmics package.

Each set of discriminating peptides was tested for over-representation about specific taxa and functions. Significant enrichments were obtained *via* the Fisher test comparing the observed vs. expected proportions of peptides in a specific category. We implemented a permutation procedure to control for multiple testing and the hierarchical nature of the microbial peptides annotation. We computed the empirical *p*-values using 10,000 permutations in which the peptides were assigned to a random category, obtaining the expected distribution under the null hypothesis of no associations. Sensitivity analysis was performed to identify the most robust results, among the identified enrichment. The whole procedure described above, from variable selection to over-representation analysis, was repeated starting from the residual of the regression of log-peptide intensities on age, sex, and the other clinical characteristics (e.g., discrimination analysis for tumor site was adjusted for age, sex, grade, stage, and TILs). We described as statistically significant the enrichments with the empirical *p*-value lower than 0.05 in the main and/or in the sensitivity analysis.

Correlation analyses were carried out starting from the results of the enrichment analyses. Specifically, for each set of microbial peptides contributing to a significant enrichment, we ran a canonical correlation analysis (CCA) against the human peptide dataset (*rCCA* function in mixOmics). Briefly, this method looks for a linear combination of the variables to reduce the number of dimensions (human peptides) maximizing the correlation with the reference matrix (microbial peptides). To simplify, the method applies the same sPLS logic to continuous outcomes, rather than to categorical values. Similarly, for each set of human peptides contributing to a significant enrichment, we ran a CCA against the microbial peptide dataset. Final, over-representation of specific taxa and functions were performed as described above.

Finally, we performed a power analysis, to provide useful information about the appropriate (minimum) sample size for metaproteomic analyses in future studies. Since it is not possible to derive an analytical formula for datasets in which the number of variables exceed the observations, we computed it empirically. We used a modified version of an algorithm^4^, initially described to generate simulated mRNA data, to simulate a metaproteomic dataset of 10,000 individuals using the observed feature distribution (the whole sample set of this study) as the reference, within which we defined two groups (5,000 individuals each), randomly. Then, we manually included 300 differentially abundant features between two groups, with effect size (log_2_FC) equal to 1.25, 1.5, 2 (100 features each). Finally, we simulated sampling from the reference dataset, for each N (sample size) within 15 and 100 individuals per group, and we generated 1,000 random subsamples of the whole dataset. For each N, the empirical power of the study was defined as the number of times the differentially abundant features were correctly identified as associated with the outcome of interest, out of the 1,000 simulations. We applied an FDR correction for multiple testing to control the type I error. The described procedure ends in a final output in which the empirical power of the study is a function of N and log_2_FC.

## Results

### Taxonomic and Functional Profile of the Tumor-Associated Colonic Luminal Metaproteome

Colonic luminal content samples (overlaying the radically resected lesion area) were collected immediately after surgery from 24 patients with CC. As luminal contents were generally comparable to feces in terms of appearance and texture, an established sample preparation pipeline set up for the metaproteomic analysis of fecal samples (Tanca et al., 2014) was employed. Samples were therefore subjected to protein extraction, FASP, and liquid chromatography/high-resolution mass spectrometry analysis to study the CC-associated colonic luminal metaproteome (i.e., the whole protein assortment of the colon lumen microenvironment, including a microbial and a human portion). To improve the identification yield (Tanca et al., 2016), a custom metagenomic database was generated by sequencing the pool of DNA extracts obtained from the same samples subjected to metaproteomic analysis, and used in parallel with publicly available sequence databases for peptide identification. Setting the FDR threshold to 1%, a total of 48,764 peptides were quantified through bioinformatic analysis of mass spectrometry data; peptide intensity data were used as quantitative measure and associated with taxonomic and functional annotations (refer to the section “Metaproteome analysis” within “Materials and Methods” for details concerning bioinformatic analysis). Based on taxonomic filtering, 29,455 peptides were classified as microbial and 8,470 as human. Complete lists of microbial and host peptides, along with their abundances and taxonomic/functional annotations, are presented in Supplementary Datasets 1, 2, respectively.

**FIGURE 1:**
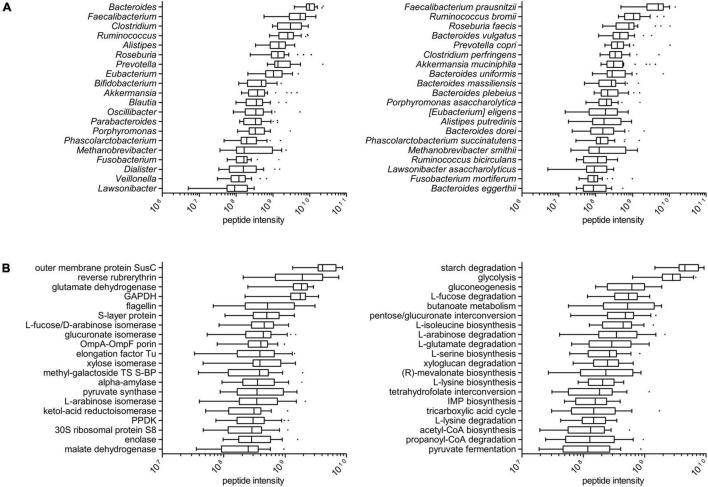
Taxonomic and functional profile of the tumor-associated colonic luminal metaproteome. **(A)** Tukey’s boxplots showing the top 20 microbial genera (left) and the top 20 species (right), ordered according to the median of the relative abundance (summed peptide intensity) distribution among patients. **(B)** Tukey’s boxplots showing the top 20 microbial functions (KOGs; left) and the top 20 pathways (right), ordered according to the median of the relative abundance (summed peptide intensity) distribution among patients.

Among the microbial peptides, 17,350 sequences could be annotated down to the genus level and 8,792 down to the species level; microbial peptides could be assigned to 186 different genera (114 on average per sample, ranging from 88 to 134) and 300 different species (178 on an average per sample, ranging from 139 to 205). As illustrated in Figure 1A, *Bacteroides* showed the highest median abundance among the genera (with *B. vulgatus*, *B. uniformis*, *B. massiliensis*, *B. plebeius*, and *B. dorei* being the main species identified in the metaproteome, in descending order), followed by *Faecalibacterium*, *Clostridium* (with *C. perfringens* as major species), and *Ruminococcus* (in particular, *R. bromii* and *R. bicirculans*). *Fusobacterium* resulted as the seventeenth most abundant genus (interestingly, with *F. mortiferum* being much more abundant than *F. nucleatum*), while the first archaeal genus was *Methanobrevibacter* (specifically, *M. smithii*). Boxplots showing taxa abundance distribution according to higher taxonomic levels (phylum and family) are provided in Supplementary Figure 1.

In functional terms, a total of 1,218 and 1,305 different protein functions could be identified in the microbial and host datasets, respectively (with 100 functions in common between the two datasets). The most abundant microbial functions detected in the tumor-associated colon lumen metaproteome were mainly involved in carbohydrate transport and metabolism, although response to stress, cell motility, and translation were also represented; furthermore, the main enzymatic functions found in the metaproteome were linked to diverse metabolic pathways, such as starch degradation, glycolysis, fucose, and arabinose degradation, as well as the biosynthesis of butyrate and amino acid (Figure 1B). The taxonomic distribution (at the genus level) of the main 50 microbial functions detected in the colonic luminal metaproteome is illustrated in Supplementary Figure 2. Of note, reverse rubrerythrin, known to be involved in the response to oxidative stress in anaerobes, was heavily produced by several clostridia (especially, *Faecalibacterium* and *Ruminococcus*); generally speaking, some functions were shared by several different members of the microbiota (e.g., glycolytic enzymes), whereas others were almost exclusive of specific genera (such as hyaluronoglucosaminidase for *Clostridium* and endoglucanase for *Ruminococcus*). On the host side (Supplementary Figure 3), the most abundant functions were involved predominantly in metabolism (proteases/peptidases and amylases) and immunity (antimicrobial peptides and immunoglobulins).

### Clinicopathological Variables Explain Part of the Colonic Luminal Metaproteome Diversity

Table 1 lists the main characteristics of the 24 patients with CC (14 males and 10 females) selected for the study and of the related tumors. The median age of the patients at the time of the diagnosis was 75 years (range: 33–88). Concerning the tumor site, 15 were right CC cases and 9 were left CC cases. Among the clinicopathological features of the tumors, we considered specifically stage, grade, and TILs. The percentages of stage I, II, III, and IV cases were 25, 25, 42, and 8%, respectively; the percentages of G1, G2, and G3 cases were 8, 63, and 29%, respectively (no G4 cases were included in the study); finally, the percentage of TILs in the cancer tissues was null in 12 cases and ranged between 5 and 60% in the remaining 12 cases (median: 10%).

**TABLE 1:** Characteristics of patients and related tumor samples selected for the study.

Sample code	Gender	Age at diagnosis	Site	Stage	Grade	TILs (%)
S061	F	75	Right colon	II	G2	0
S085	M	75	Right colon	III	G2	20
S097	F	64	Right colon	II	G3	0
S099	F	77	Right colon	III	G2	0
S103	F	62	Left colon	III	G2	0
S107	F	88	Right colon	II	G2	0
S109	F	65	Left colon	III	G2	0
S111	M	77	Right colon	I	G2	0
S119	M	76	Right colon	I	G2	5
S121	M	79	Right colon	II	G2	20
S125	M	75	Right colon	I	G3	10
S131	M	72	Right colon	I	G1	0
S135	F	87	Right colon	II	G3	50
S141	M	51	Left colon	I	G1	0
S143	M	70	Left colon	IV	G2	5
S145	F	80	Left colon	III	G3	0
S147	F	79	Right colon	III	G3	60
S151	M	75	Left colon	III	G2	5
S157	M	77	Right colon	II	G3	0
S161	M	66	Left colon	III	G2	10
S165	F	33	Left colon	IV	G3	5
S175	M	66	Left colon	III	G2	10
S181	M	74	Right colon	III	G2	0
S183	M	83	Right colon	I	G2	50

**FIGURE 2:**
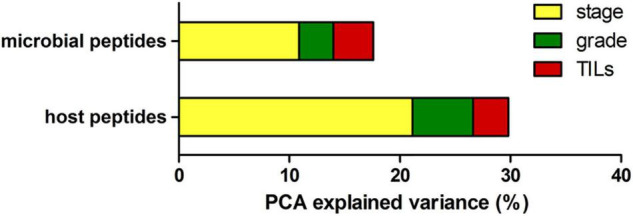
Explained variance analysis. Bar graph reporting the percentage of metaproteome variance explained by clinical variables based on PCA results, using the abundance of microbial peptides **(top)** and host peptides **(bottom)** as input data.

To investigate the proportion of variability explained by each clinicopathological variable in the whole dataset, we performed a diversity analysis *via* the PCPR2 approach (refer to the section “Statistical analyses” within “Materials and Methods” for details). As illustrated in Figure 2, 17.6% (microbial peptides) and 29.8% (host peptides) of the overall variance of the metaproteomic data could be explained by the clinicopathological variables considered in this study, with the strongest contribution provided by tumor stage (14% on average), followed by tumor grade (4%) and percentage of TILs (3%). Two sample groups were then identified for each clinicopathological variable (i.e., high- vs. low-stage samples, high- vs. low-grade samples, and TIL-positive vs. negative samples), as shown in Table 2.

**TABLE 2:** Comparisons between sample groups based on tumor-associated clinical variables.

Clinical variable	Group A	N group A	Group B	N group B
Stage	Stage I–II	12	Stage III–IV	12
Grade	G1–2	17	G3	7
TILs	TILs + (≥5%)	12	TILs − (0%)	12

We did not find significant differences (*p* < 0.05) when comparing richness (number of different peptides found) and alpha diversity (i.e., peptide diversity, according to Shannon’s index) between sample groups by applying the Wilcoxon test. Boxplots showing the distribution of richness and alpha diversity values among the sample groups are provided in Supplementary Figure 4 (microbial peptides) and Supplementary Figure 5 (host peptides).

### A Small Set of Microbial and Host Peptides Correctly Discriminate Sample Groups Based on Tumor Clinicopathological Features

We aimed at evaluating the effectiveness of colonic lumen metaproteomic data in discriminating cases with CC based on the clinicopathological characteristics of the tumor. To this purpose, we investigated the classification ability of microbial and host peptides, as well as the combined classification ability, according to the “accuracy” metric. For each tumor characteristic (stage, grade, and TILs), we identified the set of most discriminating peptides through sPLS-DA (refer to the section “Statistical analyses” within “Materials and Methods” for details).

**FIGURE 3:**
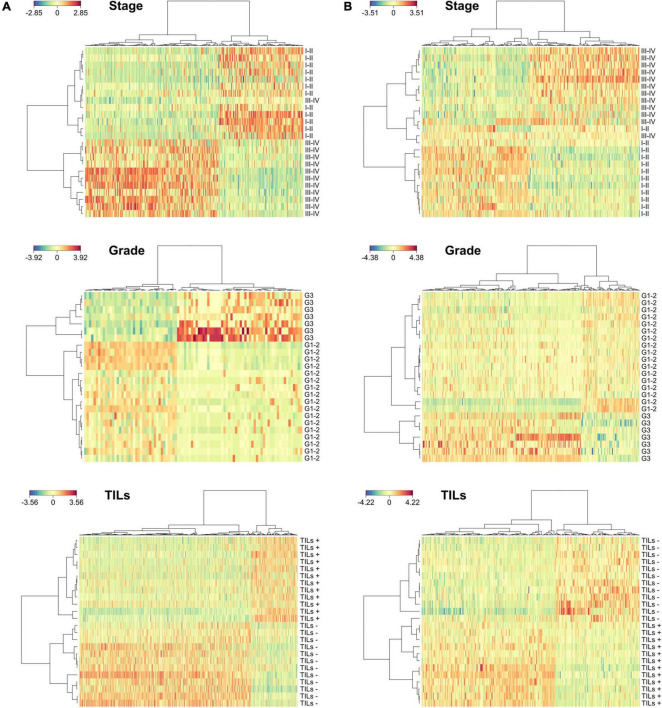
Discrimination between sample groups based on clinicopathological features according to sparse partial least squares regression discriminant analyses (sPLS-DA). **(A)** Heatmaps illustrating hierarchical clustering of sample groups based on stage (top), grade (middle), and TILs (bottom) according to microbial discriminating peptides. **(B)** Heatmaps illustrating hierarchical clustering of samples groups based on stage (top), grade (middle), and TILs (bottom) according to host discriminating peptides.

As shown in Figure 3A, we identified 294, 94, and 568 microbial peptides discriminating between stage-, grade-, and TILs-based sample groups, respectively. The accuracy was 96% for tumor stage and 100% for tumor grade and TILs (AUC > 0.99 and AUC = 1, respectively). The proportion of peptides necessary to reach the described classification performances varied from 0.27% (grade) to 1.63% (TILs). Analyzing the host metaproteome (Figure 3B), we identified 282, 301, and 290 human peptides discriminating between stage-, grade-, and TILs-based sample groups, respectively. The accuracy was 96% for stage and 100% for grade and TILs (AUC > 0.99 and AUC = 1, respectively). The proportion of peptides necessary to reach the described classification performances varied from 3.02% (stage) to 3.22% (grade). The combination of microbial and host peptides did not improve the classification performances (data not shown). Detailed data regarding the sPLS-DA are provided in Supplementary Dataset 3.

### Specific Taxa and Protein Functions Are Significantly Enriched in Peptide Sets Discriminating Colon Cancer Cases Based on Clinicopathological Features

For each set of microbial and host peptides identified in the previous analyses, we investigated for over-representation (enrichment) of specific taxonomic and/or functional features. To this aim, as detailed in the section “Statistical analyses” within “Materials and Methods”, we performed two parallel enrichment analyses for each of the sample groups listed in Table 2: a “main analysis” and a “sensitivity analysis,” with the latter also taking into account the impact of covariates (age, gender, tumor site, and clinicopathological features except the one determining a given comparison). Taxonomic and functional features significantly enriched according to both analyses (empirical *p* < 0.05) are listed in Table 3 (microbiota) and Table 4 (host). Full outputs of enrichment analyses are reported in Supplementary Dataset 4.

**TABLE 3:** Taxonomic and functional features significantly enriched in the microbial peptide sets discriminating between stage-, grade-, and TILs-based sample groups.

Taxonomic/functional level	Taxonomic/functional feature	Main analysis empirical *p*-value	Sensitivity analysis empirical *p*-value	Higher in	# peptides*
Class	Actinobacteria	<0.001	0.01	Stage III–IV	15
Order	Bifidobacteriales	<0.001	0.037	Stage III–IV	15
Family	Bifidobacteriaceae	<0.001	0.037	Stage III–IV	15
Genus	*Bifidobacterium*	<0.001	0.049	Stage III–IV	14
KOG	K01938: formate–tetrahydrofolate ligase	<0.001	0.001	Stage III–IV	5
Species	*Bacteroides fragilis*	0.01	0.007	G3	3
Species	*Bacteroides intestinalis*	0.011	<0.001	G3	3
KOG	K01784: UDP-glucose 4-epimerase	0.011	0.01	G3	2
Phylum	Bacteroidetes	0.001	<0.001	TILs −	209
Class	Bacteroidia	0.003	0.004	TILs −	200
Order	Bacteroidales	0.001	0.002	TILs −	200
Family	Bacteroidaceae	<0.001	<0.001	TILs −	149
Genus	*Bacteroides*	<0.001	<0.001	TILs −	149

*Number of discriminating peptide sequences annotated with that taxonomic/functional feature.

**TABLE 4:** Human proteins significantly enriched in the microbial peptide sets discriminating between stage-, grade-, and TILs-based sample groups.

Function	Main analysis empirical *p*-value	Sensitivity analysis empirical *p*-value	Higher in	# peptides*
K16502: cadherin-related family member 2	<0.001	0.015	Stage I–II	4
K01312: trypsin	0.001	0.001	G1–2	3
K12349: neutral ceramidase	0.003	0.004	G1–2	5
CKO-h22: prolactin-inducible protein	0.047	0.047	TILs +	3
K03911: antithrombin III	0.044	0.037	TILs +	4
K21127: protein S100-A8	0.014	<0.001	TILs +	5

*Number of discriminating peptide sequences annotated with that taxonomic/functional function.

In the microbial peptide set discriminating CC cases based on tumor stage, we found an over-representation in high-stage CC samples of a series of hierarchically related taxa, namely the lineage from Actinobacteria (class) down to *Bifidobacterium* (genus). In the same peptide set, the enzymatic function of formate–tetrahydrofolate ligase (attributable to Clostridia) was enriched as well in high-stage CC samples. When considering the host discriminant peptide set for the same comparison, cadherin-related family member 2 was enriched in low-stage CC samples.

*Bacteroides fragilis* and *Bacteroides intestinalis* were the bacterial species enriched in high-grade samples, based on the microbial peptide set discriminating the CC samples based on tumor grade. In addition, UDP-glucose 4-epimerase was the only function significantly enriched in high-grade samples. On the other hand, human trypsin and neutral ceramidase were observed as over-represented in low-grade CC samples.

Finally, we investigated the peptide sets discriminating CC cases with and without TILs. Considering microbial peptides, the lineage from Bacteroidetes (phylum) down to *Bacteroides* (genus) resulted as significantly enriched in the colonic luminal contents associated with CC tissues with no TILs. Considering the host counterpart, prolactin-inducible protein, antithrombin III, and S100-A8 were found over-represented in the lumen of TIL-positive CC samples.

We also carried out a further analysis to search for possible correlations between the main microbial peptide clusters associated with significant enrichments and host peptides. As shown in Supplementary Dataset 5, the peptide cluster assigned to the lineage Actinobacteria/*Bifidobacterium* and associated to high-stage CC cases correlated positively with a cluster of 38 host peptides, enriched in 5 protein functions (including a carbohydrate transporter and an enzyme involved in protein glycosylation), and negatively with a cluster of 44 host peptides, enriched in 2 protein functions (including alpha amylase). Furthermore, the peptide cluster assigned to the lineage Bacteroidetes/*Bacteroides* and associated with TIL-negative tumor samples correlated positively with 286 host peptides, enriched in 6 protein functions (including eosinophil peroxidase and intelectin), and negatively with 271 host peptides, enriched in other 10 protein functions (including type II keratin, catalase, and ceruloplasmin).

### Assessment of the Optimal Sample Size for Differential Metaproteomics Using Colonic Luminal Contents

**FIGURE 4:**
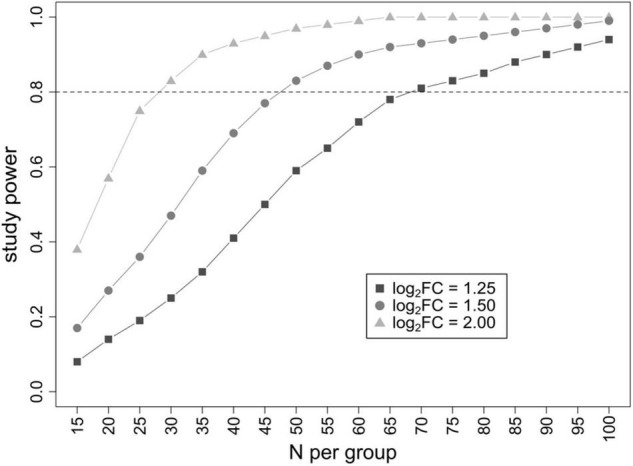
Result of power analysis. Graph plotting the study power (probability to identify differential features) as a function of the number of patients analyzed per group. Three different log_2_FC threshold values (corresponding to a small, medium, and high effect) were evaluated.

Since an analytical formula to estimate the power of the study in metaproteomic differential analyses does not exist, we have estimated it empirically on a set of 1,000 metaproteomic datasets generated *in silico*. We computed the probability to correctly identify differentially abundant features as a function of the sample size N (varying from 30 to 200 individuals per group) and the base 2 logarithm of the fold-change ratio (log_2_FC) between the two groups (small effect: log_2_FC = 1.25; medium effect: log_2_FC = 1.50; high effect: log_2_FC = 2.00), keeping the threshold of significance fixed at 0.05 after FDR correction for multiple testing. As summarized in Figure 4, a minimum of 30, 50, and 70 patients per group is needed to reach an 80% probability to identify features with true log_2_FC = 2, 1.5, and 1.25, respectively.

## Discussion

In this pilot study, we chose to analyze the luminal contents lining over CC tissues. With respect to feces, widely used as proxy for the colonic microbiota, sampling luminal contents immediately after the surgical procedure (colectomy) is expected to better preserve microbial and host metaproteomic features from changes depending on the environmental stimuli encountered at distal sites toward the rectal ampulla and, furthermore, after stool excretion. Compared to the rapid peristaltic waves in the small intestine, microbial communities and host-derived moieties composing the fecal matter are slowly propelled from cecum to rectum. Colonic microbial communities are therefore expected to be exposed for several hours to host features derived from the tumor microenvironment. Previous studies described specific differences between cecal and fecal metaproteome in animal models (Tanca et al., 2017b), reinforcing the need for a deeper investigation of microbial community functions directly sampling the colon microenvironment.

As described here, metaproteomic profiles of colonic lumen contents can provide deep information regarding both the microbial and host proteomes. In particular, the human component does not only comprise proteins from epithelial exfoliation, but also those produced and/or secreted by local immune cells. This is of special interest when dealing with inflammatory conditions, as those locally induced by CC. With respect to the microbial components, sampling and analysis of the tumor-associated microbiota during CC surgery is expected to allow for the retrieval of key microbiological information, potentially relevant to tumor management and therapy, to be associated in turn with those gathered by tumor tissue analysis routinely performed by pathology laboratories.

As most of the luminal contents collected in this study were similar to feces based on their texture, we decided to apply a protein extraction protocol previously set up for stool samples (Tanca et al., 2014). In addition, we generated a matched metagenomic database by sequencing DNA extracted from a pool of the same samples analyzed by metaproteomics, in line with the results of an earlier comparative investigation showing that the use of matched metagenomic databases (in that case obtained from human and mouse fecal samples) enables higher identification and annotation yields in gut metaproteomic studies (Tanca et al., 2016).

To the best of our knowledge, no metaproteomic characterization of human colonic luminal contents has been described to date in the scientific literature. However, among gut content samples different from stool, the mucosal-luminal interface (MLI) was sampled through endoscopic lavages and analyzed by metaproteomics in two pioneering investigations (Li et al., 2011; Presley et al., 2012); unfortunately, a limited information yield about the microbiota could be reached depending on the analytical pipelines and sequence databases available at that time. More recently, Zhang et al. (2018) used aspirate samples from colonoscopy to study the gut metaproteomic profile of children diagnosed with inflammatory bowel disease. The abundance distribution of the microbial taxa detected in the MLI samples of pediatric patients was globally comparable to that of the colonic luminal samples analyzed in this study, except for a higher rank for *Methanobrevibacter* and *Fusobacterium*, in line with previous reports, including metaproteomic data obtained from the stool of patients with CRC (Long et al., 2020). Another sample type recently subjected to metaproteomic profiling is mucus (and the related microbiota) collected from colon biopsies of patients with irritable bowel syndrome, although few details regarding microbial identifications were made available (Jabbar et al., 2021). New metaproteomic studies are thus needed to elucidate in more detail the functional differences between luminal, mucosal, and fecal microbial communities in the human gut.

The main goal of a pilot study is to demonstrate the potential of an experimental design and identifying promising trends, to be then validated in more focused and stringent investigations on a wider number of samples. Here, we aimed at evaluating the use of colonic luminal content samples to identify microbial and host proteins associated with the main clinicopathological features of CC, namely stage, grade, and TILs. The main limitation (typical for a pilot study) was the low number of samples analyzed, which could in turn lead to a low statistical power. Accordingly, we intentionally chose to apply rigorous statistical methods that are able to reduce the type I error rate (false positives), at the cost of a possibly higher type II error rate (false negatives).

First, we applied a descriptive statistical methodology based on PCA (PCPR2), commonly used for datasets with more variables than observations, to quantify the proportion of variability explained by the clinicopathological characteristics of the tumors. The results of this preliminary investigation indicate that a significant part of variability can be explained by the differences in tumor grade, stage, and TILs, supporting the likelihood to identify statistically significant peptides in differential analyses. As expected, the percentage of explained variance was slightly higher for host peptides (being derived from tumor and immune cells) compared to the microbial peptides.

Due to the low sample size of this pilot study, identification of differentially abundant peptides has been performed *via* discriminant analyses, rather than using a more stringent approach based on a differential test for each peptide followed by the correction of the *p*-values for multiple testing. To extract the most relevant features from each comparison and avoid overfitting, we employed a permutation-based procedure in which the whole dataset was randomly split into training and test set 1,000 times, keeping the features appearing in at least 50% of the permutations. The described procedure led to a minimization of the type I and type II error rates simultaneously. This approach allowed us to perfectly discriminate CC cases based on tumor grade and TILs, whereas the classification performance was slightly lower for the tumor stage. Interestingly, the only outlier in the stage-based classification (according to both microbial and human data, as illustrated in Figures 3A and B, respectively) correspond to the same patient (S165), who is a young-onset CC case (with an age significantly lower compared to all the other patients). Young-onset CC is known to have peculiar clinicopathological characteristics (Akimoto et al., 2021), including a possibly different associated microbiota (Yang et al., 2021). This might reasonably explain the behavior of that patient as an outlier.

After identifying the most relevant peptides for each comparison, we investigated over-representation of specific taxonomic and functional features through enrichment analysis. Again, in these analyses, we applied a permutation-based procedure to reduce type I and type II errors as much as possible. Specifically, we assigned each peptide to a random taxonomic/functional category 10,000 times to estimate the null distribution expected under the hypothesis of no associations (null hypothesis). The comparison of observed vs. expected distribution provided the list of empirical *p*-values for the enrichment analysis. We further reduced the type I error rate performing a sensitivity analysis, in which the described procedure was repeated after removing the effect of important sample characteristics (residuals from a multivariate linear regression analysis). Enrichment analysis enabled us to identify several microbial taxa and functions, as well as human proteins, as significantly enriched in the discriminating peptide sets based on the clinicopathological features of CC. Considering tumor stage, the lineage from Actinobacteria (class) down to *Bifidobacterium* (genus) was enriched in the colonic metaproteome associated to high-stage tumor samples. *Bifidobacterium* is known for its potential role in preventing CRC, possibly due to the enhancement of the inflammation-suppressive function of Tregs (O’Callaghan and van Sinderen, 2016; Asadollahi et al., 2020; Sun et al., 2020); however, a different interplay between bifidobacteria and carcinoma cells might occur after the establishment of CRC. Lactic acid and acetate produced by bifidobacteria, as well by other members of the gut microbiota, can serve as an energy source for carcinoma cells, supporting tumor progression. Noteworthy, a recent study based on over 1,000 CRC cases found a strong association between bifidobacteria in CRC tissues and malignant signet cells, a known indicator of poor prognosis (Kosumi et al., 2018). Of note, peptides discriminating CC cases based on stage were also significantly enriched for the taxonomic lineage from Euryarchaeota (phylum) to Methanobacteriaceae (family), even though only according to the “sensitivity analysis” (refer to Supplementary Dataset 4). Methanobacteriaceae have been already described as more abundant in the metaproteome of patients with CRC compared to healthy controls (Long et al., 2020); specifically, an active involvement of *Methanobrevibacter* in CRC carcinogenesis has been hypothesized (Alomair et al., 2018). In addition, we found formate–tetrahydrofolate ligase produced by the colonic microbiota (specifically by Clostridia) as significantly enriched in high-stage tumors. Formate is generated by members of the GM during anaerobic fermentation and can be also produced by the host; it can, in turn, become a substrate for the growth of both aerobic and anerobic bacteria or enter the circulation, being used by almost all the tissues for the synthesis of nucleotides, that is essential during tumor growth (Pietzke et al., 2020). Since some studies reported an important formate demand in CRC, as inferred from the high expression of the enzyme MTHFD1L involved in its production (Agarwal et al., 2019; He et al., 2020), we may hypothesize a competition for formate between CRC and GM. To the best of our knowledge, no specific metabolomic investigations aiming at measuring formate in gut contents or in feces of patients with CC and/or healthy controls have been described so far. Therefore, further studies are needed to verify this hypothesis and elucidate possible relationships among formate/tetrahydrofolate metabolism, colonic microbiota, and CC. Moving to host proteins, cadherin-related family member 2, also named protocadherin LKC, found in this study as significantly enriched in low-stage tumors, had been proposed in the past as a potential CC suppressor by its ability to induce contact inhibition of cell proliferation (Okazaki et al., 2002). Furthermore, lactotransferrin and peroxiredoxin-2 were found as human functions significantly enriched in high-stage CC cases, even if according to the “sensitivity analysis” only. The direct correlation between lactotransferrin protein content and the stage of the disease has been previously demonstrated in metastatic CRC tissues (Burlaka et al., 2019). Furthermore, lactotransferrin was found as gradually increased in non-adenomatous colon polyp, non-metastatic CC, and metastatic CC tissues when compared to the normal colon (Saleem et al., 2019). Peroxiredoxin-2 upregulation was reported to correlate with CRC progression (Peng et al., 2017).

As far as tumor grade classification is concerned, we found that *Bacteroides fragilis* was significantly enriched in the colonic metaproteome of high-grade cases. This bacterial species has been found as more abundant in the CRC microbiota compared to that of the healthy controls in numerous studies, and its toxin has been hypothesized to promote colon carcinogenesis (Boleij et al., 2015; Dai et al., 2018; Haghi et al., 2019; Thomas et al., 2019; Chen et al., 2021). Furthermore, among host functions, trypsin and neutral ceramidase were observed as significantly enriched in low-grade tumors. Trypsin expression in CRC has been associated with unfavorable clinicopathological features and reduced survival (Yamamoto et al., 2003; Ziapour et al., 2011). Neutral ceramidase was reported to having a role in the development of CC through antiapoptotic and proliferative processes (García-Barros et al., 2016).

The prognostic effect of TILs related to CRC has been increasingly recognized, as a stronger lymphocytic reaction has been associated with longer patient survival, particularly in patients with stage III and right-sided tumors (Ropponen et al., 1997; Huh et al., 2012). The application of a “TILs + vs. TILs−” classification adopted in this study was based on both statistical and biological reasons. In statistical terms, this classification allowed us to obtain groups of identical size; under a biological perspective, we can expect that even a very low degree (e.g., 5%) of lymphocyte infiltration might exert a significant effect on the functional dynamics of the tumor-associated microbiota and should not be included in the same group of cases without detectable TILs. We also evaluated an alternative classification, more similar to those that have recently begun to be used in the clinical practice for prognostic aims (Pagès et al., 2018; Taube et al., 2018), based on which the percentage of cases with low (<10%) and intermediate-high (≥10%) TIL level were 67 and 33%, respectively. As the number of differential features identified based on the “TILs + vs. TILs −” classification was much higher compared to the alternative one (data not shown), we decided to focus on the former. Among the features differentially enriched in the peptide sets discriminating based on TILs and higher in TILs cases, we found the lineage the lineage from Bacteroidetes (phylum) down to *Bacteroides* (genus). Bacteroidaceae and *Bacteroides* were found as significantly more abundant in the fecal microbiota of patients with CRC when compared to healthy controls (Yang et al., 2019). In addition, *Bacteroides* spp. are able to degrade glycans and, depending on specific dietary patterns, some strains of *Bacteroides* can act as mucus degraders, causing a reduction in the thickness of the mucus layer (Desai et al., 2016); this reduction can in turn lead to CC development (as demonstrated in mice; Velcich et al., 2002) or to intestinal inflammation (Larsson et al., 2011). Among host functions significantly enriched in the TILs + group, antithrombin III has been found in lower amount in the blood serum of patients with CRC compared to the healthy controls (Peltier et al., 2016). Another host function that correlates with TIL (even if according to sensitivity analysis only) is cadherin 17, a calcium-dependent transmembrane glycoprotein playing a role in cell-cell adhesion and expressed by the intestinal epithelium (Su et al., 2008). Findings regarding the involvement of this protein in colon carcinogenesis are ambiguous, since it has been found underexpressed in human CRC with a role in maintaining the intestinal homeostasis (Chang et al., 2018), overexpressed in metastatic human CC cells (Bartolomé et al., 2014), as well as associated with cell proliferation, metastasis, and poor survival in CRC (Tian et al., 2018). Further, protein S100-A8 has been reported to facilitate CRC migration and differentiation (Duan et al., 2013; Zha et al., 2016).

Furthermore, we decided to carry out a power analysis, based on the results from this pilot study, to determine the minimum sample size needed to identify a satisfactory number of differential features. Being aware of the limitations induced by the low sample size, we estimated the number of samples needed in future studies to detect significant results in differential analyses based on the observed distribution of peptides, varying the effect size of the association, and considering type I error rate equal to 0.05 (after adjustment for multiple comparisons). Again, we estimated it empirically, since it is not possible to derive an analytical formula for a dataset with more variables than observations.

Other limitations of the experimental design of this pilot study might be the absence of control (healthy) and colon adenoma (precancerous lesion) samples, as well as of information regarding the survival rate and response of patients to therapy. Nevertheless, it is worth noting that this investigation was not specifically aimed at identifying diagnostic and/or prognostic biomarkers. In addition, we cannot rule out that part of the metaproteomic data variance could be explained by diet; however, no information about patients’ dietary habits were available.

In conclusion, this study describes an extensive characterization of the tumor-associated colonic luminal content metaproteome, together with the pilot investigation of how the metaproteomic profile changes with respect to tumor clinicopathological features (stage, grade, and TILs). Of note, we were able to discriminate tumor characteristics using a relatively low number of peptides, suggesting the potential of metaproteomic studies in the identification of prognostic biomarkers for CC progression and survival. Several microbial taxa (including *Bifidobacterium*, *B. fragilis*, and *B. intestinalis*) were significant enriched in the peptide sets discriminating between high- and low-stage (or -grade) tumors. Moreover, peptides functionally assigned to formate–tetrahydrofolate ligase exhibited higher abundance in high-stage cancer tissues. Finally, we performed a power analysis enabling the assessment of the optimal sample size for a differential metaproteomic study using colonic luminal contents.

In perspective, future studies with higher numbers of patients and complete follow-up information are needed to verify the clinical potential of these data in the definition of new prognostic indicators, to investigate the combined effects of tumor stage/grade/TILs, and to validate their value in the understanding of CC progression. In addition, further developments and standardization of metaproteomic protocols are expected to improve the quality, reproducibility, and robustness of metaproteomic data in the near future.

## Data Availability Statement

The datasets presented in this study can be found in online repositories. The names of the repository/repositories and accession number(s) can be found below: http://www.proteomexchange.org/, PXD017467.

## Ethics Statement

The studies involving human participants were reviewed and approved by the Bioethics Committee of the “Azienda Sanitaria Locale di Sassari” (n. 2032/CE, 13/05/2014). The patients/participants provided their written informed consent to participate in this study.

## Author Contributions

SU and AT designed the study. GP, MD, MM, AS, and AP collected the samples and clinical data. PC-R and AM performed histopathological evaluation of tumors. AT and GP performed sample preparation for metaproteomic analysis. MA and RS performed sample preparation for metagenome sequencing. AT and MA conceived and performed data analysis. GF conceived and performed statistical analyses. AT, SU, MA, and GF drafted the manuscript. MD and PC-R critically revised the manuscript. All authors read and approved the final version of the manuscript.

## Conflict of Interest

The authors declare that the research was conducted in the absence of any commercial or financial relationships that could be construed as a potential conflict of interest.

## Publisher’s Note

All claims expressed in this article are solely those of the authors and do not necessarily represent those of their affiliated organizations, or those of the publisher, the editors and the reviewers. Any product that may be evaluated in this article, or claim that may be made by its manufacturer, is not guaranteed or endorsed by the publisher.

## Footnotes

1
ftp://ftp.cngb.org/pub/SciRAID/Microbiome/humanGut_9.9M/GeneCatalog/IGC.pep.gz

2
https://unipept.ugent.be

3
https://biit.cs.ut.ee/clustvis_large

4
https://github.com/ConesaLab/MOSim
