# Supplementary material for: Metaproteomic Profile of the Colonic Luminal Microbiota From Patients With Colon Cancer
Source: Front Microbiol. 2022 Apr 14;13:869523. doi: 10.3389/fmicb.2022.869523 (PMC9048685; doi:10.3389/fmicb.2022.869523)
Supplement: Supplementary file 1 [file Presentation_1.PDF]

Supplementary Material

Metaproteomic profile of the colonic luminal microbiota from colon cancer patients

Alessandro Tanca[1], Marcello Abbondio[1], Giovanni Fiorito[1,2], Giovanna Pira[1], Rosangela Sau[1], Alessandra Manca[3], Maria Rosaria Muroni[4], Alberto Porcu[4], Antonio Mario Scanu[4], Paolo Cossu-Rocca[4,5], Maria Rosaria De Miglio[4], Sergio Uzzau[1*]

[1]Department of Biomedical Sciences, University of Sassari, Sassari, Italy

[2]MRC Centre for Environment and Health, Imperial College London, London, UK

[3]Department of Pathology, AOU Sassari, Sassari, Italy

[4]Department of Medical, Surgical and Experimental Sciences, University of Sassari, Sassari, Italy

[5]Department of Diagnostic Services, Surgical Pathology Unit, "Giovanni Paolo II" Hospital, ASSL Olbia-ATS Sardegna, Olbia, Italy

Content:

Supplementary Dataset Legends: 5

Supplementary Figures (and Legends): 5

1 Supplementary Data

Supplementary Dataset 1. List of the microbial peptides quantified in the study, with their corresponding taxonomic/functional annotation and abundance data; list of phyla, families, genera, species, KOGs and pathways identified in the study, with the corresponding abundance data.

Supplementary Dataset 2. List of the human peptides quantified in the study, with their corresponding functional annotation and abundance data; list of KOGs identified in the study, with the corresponding abundance data.

Supplementary Dataset 3. Lists, metrics, and annotations of microbial and host discriminating peptides found by sPLS-DA.

Supplementary Dataset 4. Significant results of enrichment analyses (main analysis of microbial peptides; sensitivity analysis of microbial peptides; main analysis of host peptides; sensitivity analysis of host peptides).

Supplementary Dataset 5. Correlations between two of the main microbial peptide clusters associated to significant enrichments (see Table 3) and host peptides, with relative significantly enriched functions.

2 Supplementary Figures and Tables

2.1 Supplementary Figures

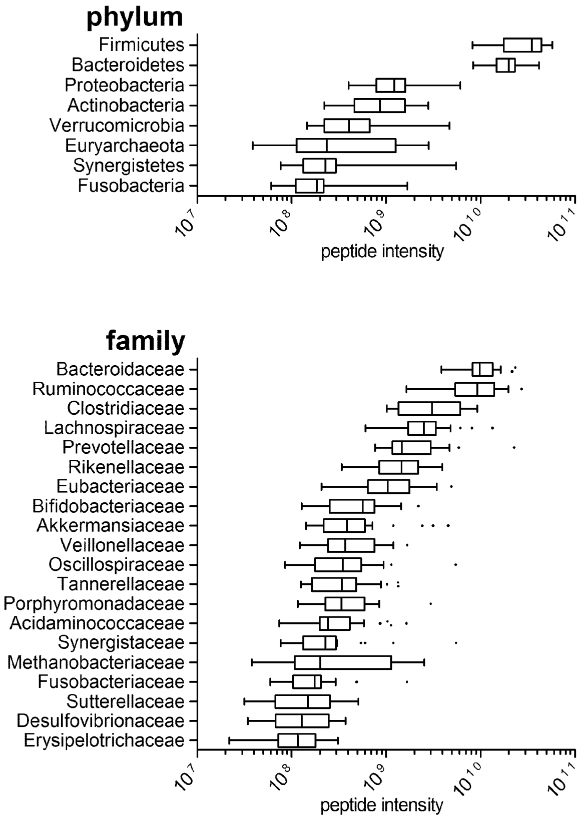

Supplementary Figure 1. Tukey's boxplots showing the top 8 microbial phyla (top) and the top 20 microbial families (bottom) in the tumor-associated colonic luminal metaproteome. Taxa are ordered according to the median of the relative abundance (summed peptide intensity) distribution among patients.

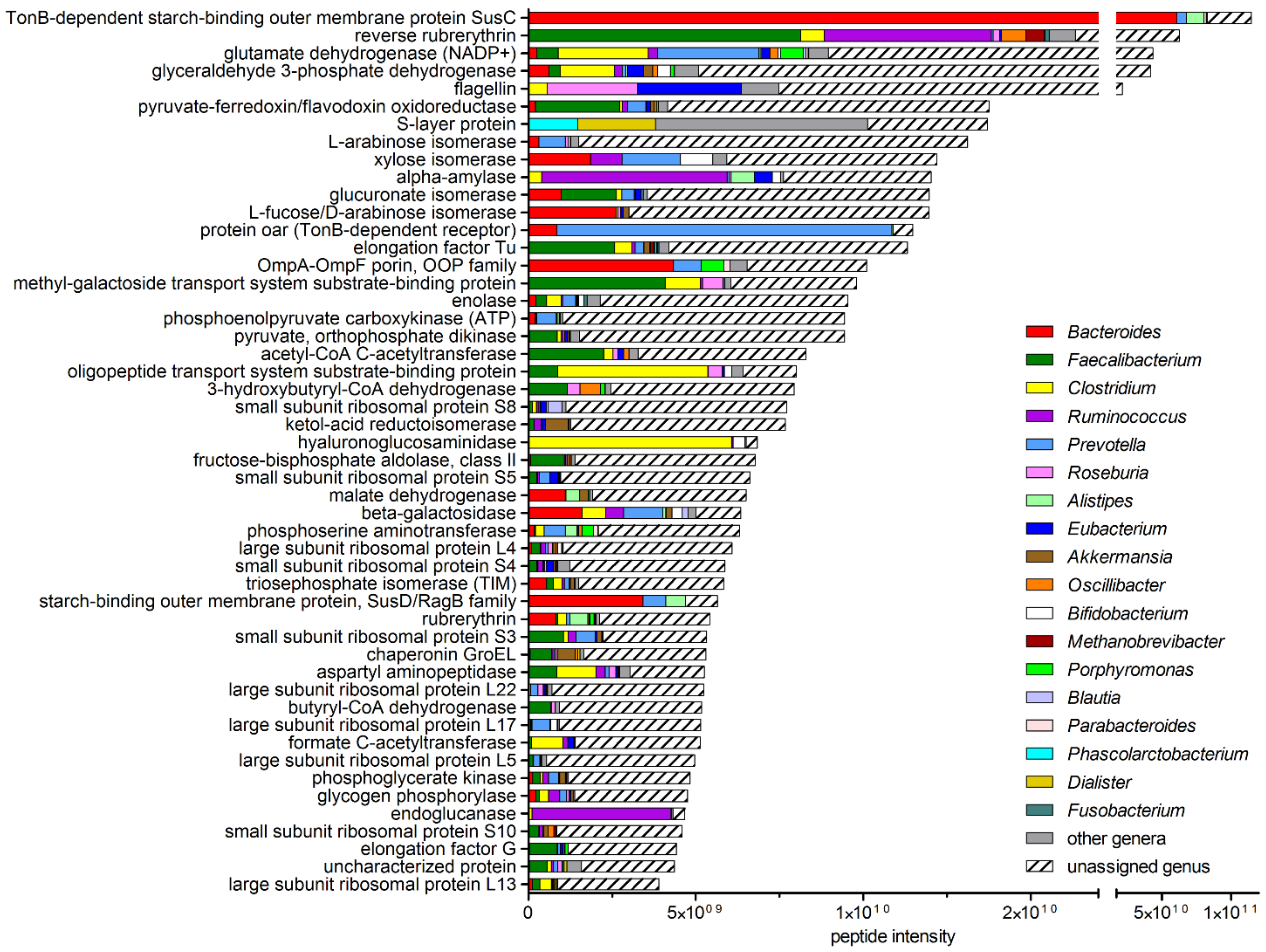

Supplementary Figure 2. Taxonomic distribution (at the genus level) of the main 50 microbial functions (KOGs) detected in the colonic luminal metaproteome. Functions are ordered according to the total abundance (summed peptide intensity) measured in the study samples.

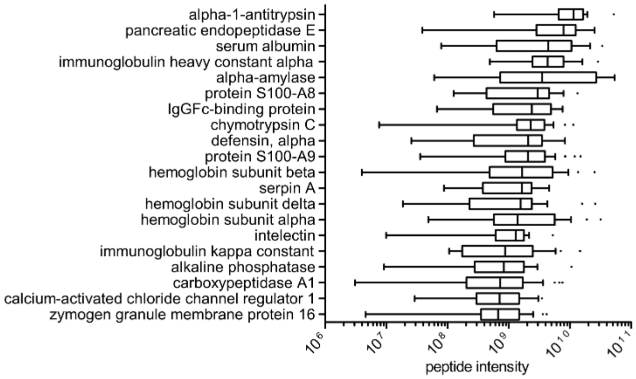

Supplementary Figure 3. Tukey's boxplots showing the top 20 human proteins detected in the tumor-associated colonic luminal metaproteome. Proteins are ordered according to the median of the relative abundance (summed peptide intensity) distribution among patients.

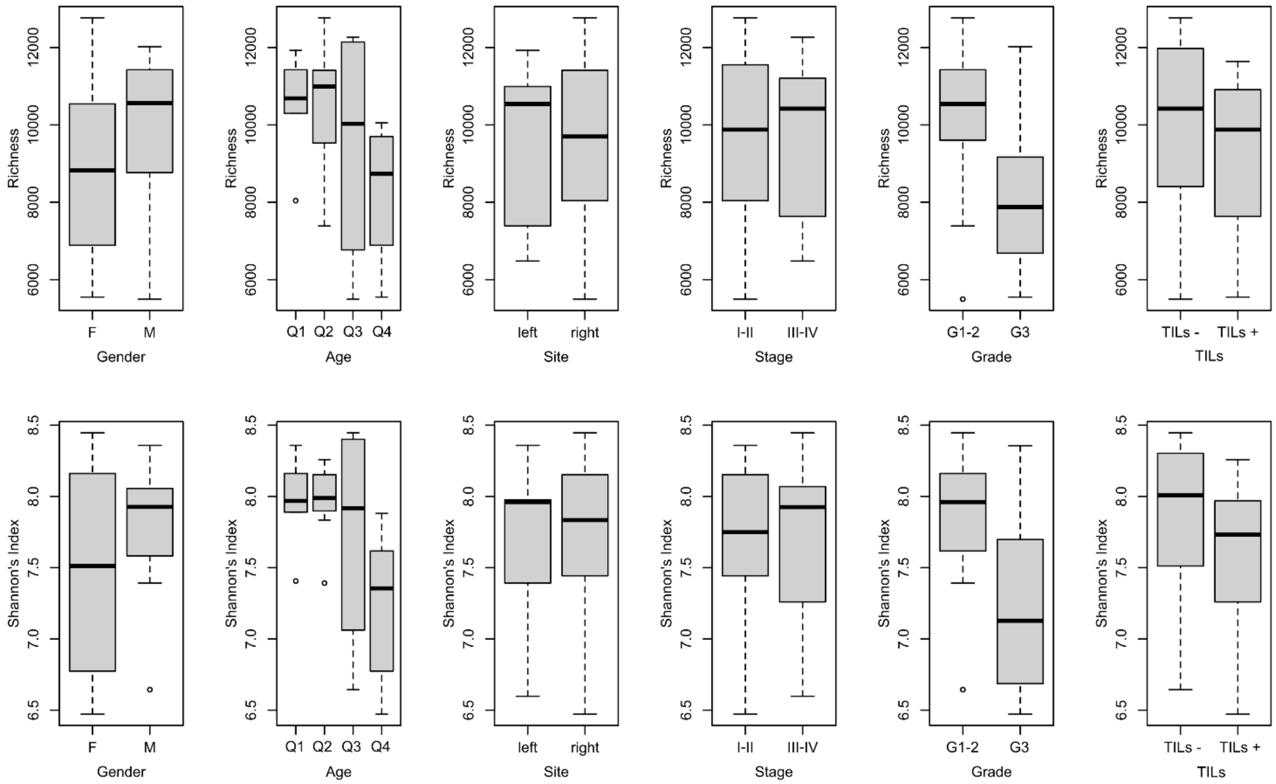

Supplementary Figure 4. Boxplots showing distribution of richness (top) and alpha diversity (calculated according to Shannon's index; bottom) in sample groups based on microbial peptide abundances.

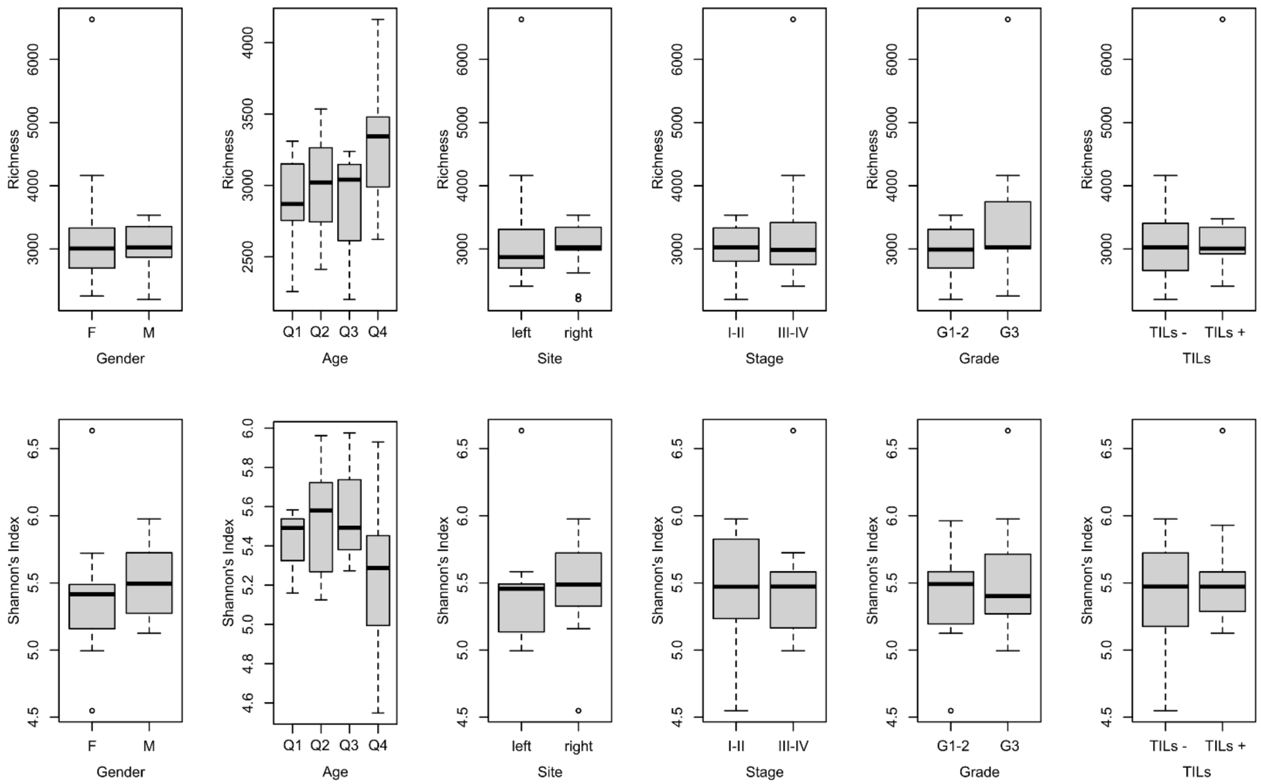

Supplementary Figure 5. Boxplots showing distribution of richness (top) and alpha diversity (calculated according to Shannon's index; bottom) in sample groups based on host peptide abundances.
